# d-enantiomers of CATH-2 enhance the response of macrophages against *Streptococcus suis* serotype 2

**DOI:** 10.1016/j.jare.2021.05.009

**Published:** 2021-05-26

**Authors:** Roel M. van Harten, Johanna L.M. Tjeerdsma-van Bokhoven, Astrid de Greeff, Melanie D. Balhuizen, Albert van Dijk, Edwin J.A. Veldhuizen, Henk P. Haagsman, Maaike R. Scheenstra

**Affiliations:** a Division of Infectious Diseases and Immunology, Department of Biomolecular Health Sciences, Utrecht University, Utrecht, the Netherlands; b Wageningen Bioveterinary Research, Wageningen University & Research, Houtribweg 39, 8221 RA Lelystad, the Netherlands

**Keywords:** Cathelicidin, CATH-2, *Streptococcus suis*, Macrophage, Mouse

## Abstract

**Introduction:**

Due to the increase of antibiotic resistant bacterial strains, there is an urgent need for development of alternatives to antibiotics. Cathelicidins can be such an alternative to antibiotics having both a direct antimicrobial capacity as well as an immunomodulatory function. Previously, the full d-enantiomer of chicken cathelicidin-2 (d-CATH-2) has shown to prophylactically protect chickens against infection 7 days post hatch when administered *in ovo* three days before hatch.

**Objectives:**

To further evaluate d-CATH-2 in mammals as a candidate for an alternative to antibiotics.

In this study, the prophylactic capacity of d-CATH-2 and two truncated derivatives, d-C(1–21) and d-C(4–21), was determined in mammalian cells.

**Methods:**

Antibacterial assays; immune cell differentiation and modulation; cytotoxicity, isothermal titration calorimetry; *in vivo* prophylactic capacity of peptides in an *S. suis* infection model.

**Results:**

d-CATH-2 and its derivatives were shown to have a strong direct antibacterial capacity against four different *S. suis* serotype 2 strains (P1/7, S735, D282, and OV625) in bacterial medium and even stronger in cell culture medium. In addition, d-CATH-2 and its derivatives ameliorated the efficiency of mouse bone marrow-derived macrophages (BMDM) and skewed mouse bone marrow-derived dendritic cells (BMDC) towards cells with a more macrophage-like phenotype. The peptides directly bind lipoteichoic acid (LTA) and inhibit LTA-induced activation of macrophages. In addition, *S. suis* killed by the peptide was unable to further activate mouse macrophages, which indicates that *S. suis* was eliminated by the previously reported silent killing mechanism. Administration of d-C(1–21) at 24 h or 7 days before infection resulted in a small prophylactic protection with reduced disease severity and reduced mortality of the treated mice.

**Conclusion:**

d-enantiomers of CATH-2 show promise as anti-infectives against pathogenic *S. suis* for application in mammals.

## Highlights

• D-CATH-2 has strong antimicrobial activities towards multiple S.suis strains.
• D-CATH-2 ameliorates macrophage function.
• DCATH-2 binds LTA.
• DCATH-2 has prophylactic effect against S. *suis* infection in vivo.

## Graphical abstract

**Figure:**
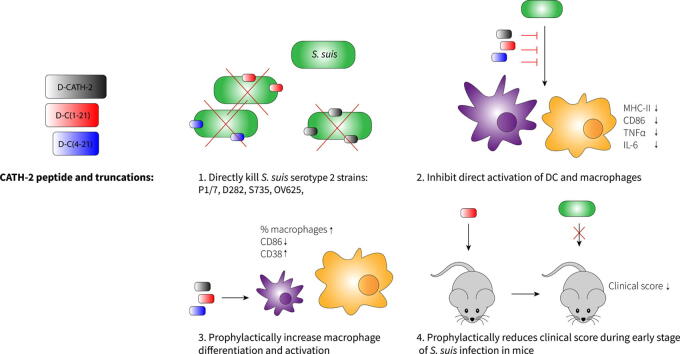

## Introduction

Since the discovery of penicillin in 1928, antibiotics have saved billions of lives around the world [1]. However, due to emergence of antibiotic resistance, the development of novel antibiotics is urgently needed [2]. A promising alternative to antibiotics are host defense peptides (HDPs). Cathelicidins are an important family of HDPs, which play an important role in the innate immune response to infections [3], [4], [5]. Cathelicidins are characterized by their highly conserved precursor cathelin-domain, but the active, mature peptides are highly variable in sequence and structure [4], [6]. They are strongly upregulated during infection [7], [8] and despite their variable sequence, almost all cathelicidins show strong antimicrobial activity against many different bacteria [9], viruses [10], [11], fungi [12], [13], and parasites [14], [15]. This antimicrobial activity is based on electrostatic membrane interactions, which makes resistance development less likely, as lipid targets can not easily mutate. In addition, many peptides have intracellular targets as well [16]. Besides their direct multivalent antimicrobial activities, the immunomodulatory functions of these peptides makes them of special interest for potential clinical applications [5].

One of the best studied immunomodulatory functions of cathelicidins is neutralization of the capsular structures lipopolysaccharides (LPS) or lipoteichoic acid (LTA). These capsular structures decorate the outer membrane of Gram-negatives or the peptidoglycan layer of Gram-positives, respectively, and induce potent immune responses through TLR-4 or TLR-2, which leads to strong overactivation in sepsis patients [17]. Cathelicidins can prevent this overstimulation of the immune system [9], [18]. Besides this inhibition of LPS- and LTA-induced cell activation, cathelicidins can also enhance the uptake of DNA and thereby increase TLR-9 activation, [19], [20], [21] induce chemokine and cytokine release, and are involved in phagocytosis [22]. In addition, cathelicidins can skew macrophage differentiation towards a more pro-inflammatory phenotype [23]. Taken together, the indirect immune modulatory effects might contribute more to protection against infections than the direct antimicrobial activity. Injection of cathelicidins in chicken eggs three days before hatch, reduced the morbidity and bacterial load when the chickens were infected seven days post hatch. The low concentration of peptide and the long period of time between administration and infection suggests that the immunomodulatory activity of CATH-2 exerted the protective effect [24].

*Streptococcus suis* (*S. suis*) is a Gram-positive facultative anaerobe bacterium that is found in almost all pigs as a commensal of the respiratory microbiota. It can also cause invasive infections in young piglets, such as sepsis, meningitis, endocarditis, and may cause sudden death [25]. In addition, *S. suis* is a zoonotic agent that can cause sepsis and meningitis in humans and is the prevalent cause of meningitis in several Southeast Asian countries [26]. The bacterium is encapsulated with a large polysaccharide capsule to prevent phagocytosis-dependent clearing [27]. Up to 35 various capsular serotypes of *S. suis* have been identified so far, of which serotype 2 is found most often in diseased pigs worldwide, followed by serotype 9 and 3, although regional differences in prevalence do occur. In human cases serotype 2 is the most prevalent [28]. Many *S. suis* strains carry resistance genes, most likely introduced due to prophylactic use of antibiotics in livestock industry [28], [29].

To determine putative application of cathelicidins in prevention and treatment of *S. suis* infections in mammals, the direct antibacterial capacity of the full d-enantiomer of chicken cathelicidin-2 (d-CATH-2) and two derivatives were determined. In addition, the capacity of d-CATH-2 and its derivatives to skew mouse bone marrow-derived dendritic cells towards a more macrophage-like phenotype was assessed. The peptides were tested whether they could bind LTA and thereby inhibit LTA-induced activation. In addition, d-CATH-2 and its derivatives were examined for *S. suis* killing without inducing an excessive immune reaction upon infection. Finally, mice were injected with the d-CATH-2 truncated derivative d-C(1–21) 24 h before *S. suis* infection, to observe effects on the immune response and disease severity caused by *S. suis.*

## Material and Methods

### Peptides, bacterial strains and experimental animals

The 26 amino acid full d-enantiomer of chicken CATH-2 (RFGRFLRKIRRFRPKVTITIQGSARF-NH_2_) (d-CATH-2) with a net charge of 9+ and two truncated derivatives (d-C(1–21), 8+ and d-C(4–21), 7+) were used in this study. The peptides were synthesized by Fmoc-chemistry at China Peptides (CPC scientific, Sunnyvale, CA, USA) and purified by reverse phase high-performance liquid chromatography to a purity of >95%. Lyophilized peptides were dissolved in endotoxin free water.

*S. suis* serotype 2 strains P1/7, D282, S735, and OV625 were used in this study. All strains have been previously characterized [30]. Bacterial strains were grown overnight at 37 °C from glycerol stocks in Todd-Hewitt broth (THB, Oxoid Ltd., London, UK) before use.

Seven to ten week old Crl:CD-1 mice (both male and female) were purchased from Charles River (Germany). All mice were kept under specific pathogen-free conditions with free access to food and water under the guidelines for animal experimentation as approved by the Dutch central authority for scientific procedures on animals (CCD, License number: AVD108002015175).

### Antibacterial activity

*S. suis* serotype 2 strains P1/7, D282, S735, and OV625 were grown into mid-logarithmic phase for 3–4 h at 37 °C in THB, after which bacteria were centrifuged at 1200×*g* for 10 min at 4 °C and resuspended in fresh THB. Bacterial concentration was determined by measuring optical density at 620 nm with an OD of 1.0 being equivalent to 1x10^8^ colony forming units (CFU)/mL. 1x10^6^ CFU/mL *S. suis* was mixed with different concentrations of d-CATH-2 and derivatives (0.63 – 40 µM) and incubated for 3 h at 37 °C. Ten-fold dilutions were prepared and spread in Tryptic Soy agar (TSA) plates containing 5% (v/v) defibrinated sheep blood (Oxoid) and colonies were allowed to grow for 48 h. Minimal Bactericidal Concentration (MBC) was defined as ≤100 CFU/mL (2 log CFU/mL), the detection limit of the assay.

### Cell culture and flow cytometry

Murine bone marrow cells, isolated from the femur and tibia of both hindlegs, were stored in fetal calf serum (FCS) (Corning, NY, USA) containing 10% DMSO (Sigma-Aldrich, MO, USA) in liquid nitrogen. Cells were grown at a concentration of 5x10^5^ cells/mL in RPMI-1640 without phenol red (Thermo Fisher Scientific, MA, USA) supplemented with 10% FCS and 1% penicillin/streptomycin (Thermo Fisher Scientific). Bone marrow-derived macrophages (BMDM) and bone marrow-derived dendritic cells (BMDC) were cultured by adding 20 ng/mL murine recombinant M−CSF or GM-CSF (PeproTech, NJ, USA) respectively. Where indicated, cells were supplemented with 1.25 µM peptide at day 1, which was replaced by fresh medium at day 2. The medium of all cells was replaced by fresh medium without antibiotics at day 3. At day 6 cells were stimulated with 1 µg/mL LTA from *S. aureus* (LTA-SA) (Invivogen, CA, USA) or with the different *S. suis* strains at a multiplicity of infection (MOI) of 0.2. Medium containing *S. suis* was removed after 2 h and replaced by medium containing 200 µg/mL gentamycin (Sigma) and left for an additional 22 h. After 24 h, medium was collected and stored at −20 °C for cytokine measurements. Cells were incubated for 5 min with 0.5 mM EDTA in PBS after which they were resuspended by vigorous pipetting and used for flow cytometry. Cells were resuspended in flow cytometry buffer (PBS/0.5% BSA, Sigma) and kept on ice during the whole procedure. Cells were stained with antibodies (Table 1) for 20 min, washed and measured using aBD FACSCanto-II (BD Biosciences, NJ, USA) and analyzed with FlowJo software (Ashland, OR, USA).

**Table 1:** Antibodies.

Antigen	Clone	Label	Manufacturer
MHC-II	M5/114.15.2	FITC	eBioscience
CD11c	HL3	PE	BD Biosciences
Sirp-α	P84	PerCP-eFluor710	eBioscience
CD19	1D3	PE-Cy7	BD Biosciences
CD8α	53–6.7	APC	BD Biosciences
CD11b	M1/70	APC-Cy7	BD Biosciences
CD24	M1/69	eFluor450	eBioscience
CD86	GL-1	PerCP	BioLegend
F4/80	BM8	APC	eBioscience
Ly6C	HK1.4	eFluor450	eBioscience
CD4	RM4-5	AF488	BD Pharmingen
CD62L	MEL-14	PE	eBioscience
CD335	29A1.4	PerCP-Cy5.5	BD Biosciences
CD44	IM7	PE-Cy7	BD Biosciences
CD3e	145-2C11	APC-Cy7	BD Pharmingen
CD25	eBio3C7	eFluor450	eBioscience

Antibodies used for flow cytometry. All antibodies were diluted 1000x in flow cytometry buffer prior to use (CD19 and CD335 were used in a 500x dilution).

### Splenocyte activation

Mice were killed using CO_2_ suffocation after which the spleens were harvested. Spleens were digested with digestion buffer (1.5 WU/mL liberase TL grade, 100 Units/mL recombinant DNAse I, both Roche, Basel, Switzerland) for 30 min at 37 °C and meshed through a 40 µm filter (BD Biosciences) to prepare a single cell solution using PBS/0.5 mM EDTA wash buffer. The red blood cells were lysed using an isotonic ammonium chloride buffer (155 mM NH_4_Cl, 10 mM KHCO_3_, 0.1 mM EDTA) for 5–10 min on ice, washed 1x with PBS, after which the cells were counted and resuspended in high glucose DMEM (Thermo Fisher scientific) supplemented with 10% FCS. 5x10^5^ splenocytes were added per well in a U-bottom 96-wells plate (Corning). Total splenocytes were stimulated with 1 µg LTA-SA or the different *S. suis* strains at an MOI of 0.2. After 2 h, the supernatant was collected (by centrifugation at 700x *g* for 2 min) and the cells were resuspended in 100 µL fresh medium supplemented with 200 µg/mL gentamycin and left for an additional 22 h. After 24 h, medium was collected and stored at −20 °C for cytokine measurements.

### Cell viability and activity

WST-1 reagent (Roche) was used for determination of the metabolic activity and thereby cell viability of BMDCs and BMDMs as well as for cell activity of activated splenocytes. In both cases, 100 μL fresh medium containing 10% WST-1 was added to the cells and incubated at 37 °C. After 30–60 min, colorimetric changes were measured at 450 nm using a FLUOstar Omega microplate reader (BMG Labtech GmbH, Ortenberg, Germany). The metabolic activity is depicted as a percentage with the untreated BMDCs/BMDMs or unstimulated splenocytes set to 100%.

### ELISA

TNFα, IFNγ, IL-1β, and IL-6 were measured in supernatant (diluted in PBS/1% BSA if needed) using a Duoset ELISA kit (R&D systems, MN, USA). ELISAs were performed according to the manufacturer’s instructions. Colorimetric changes were measured at 450 nm using a FLUOstar Omega microplate reader (BMG) with a correction for background signal at 570 nm.

### NO production

NO production in the supernatant was measured using the Griess assay. 50 µL undiluted supernatant or standard was mixed with 50 µL 5% phosphoric acid (Sigma Aldrich) and 1% sulfanilamide (Merck, NJ, USA) and incubated for 5 min in the dark. Subsequently, 50 µL 0.1% N-(1-Naphthyl)ethylenediamine (NED (MERCK)) was added and left for an additional 5 min in the dark. Colorimetric changes were measured at 550 nm using a FLUOstar Omega microplate reader (BMG) and plotted to the standard curve in a 4-parameter fit.

### Isothermal calorimetry (ITC)

The interaction between the d-CATH-2 peptides and LTA-SA was tested using isothermal titration calorimetry (ITC). All ITC experiments were performed on a Low Volume NanoITC (TA instruments - Waters LLC, New Castle, USA). Peptide solution or 37.2 µM LTA-SA was prepared in MilliQ:dPBS (Gibco) in a 3:1 ratio. The chamber was filled with 164 µL LTA-SA and the peptide was loaded in the 50 µL syringe. Every 300 s, 1.99 µL peptide was titrated into the chamber at 37 °C. Data was analyzed using the Nano Analyze software (TA instruments-Waters LLC). The data of three experiments was averaged and an independent model was used to determine the peptide-LTA interaction.

### *In vivo* infection experiment

Upon arrival, mice were allowed to acclimatize for at least 7 days before the start of the experiment. The experiment was performed as depicted in Fig. 5**A.** The experiment was repeated twice to obtain in total 4 mice in the control groups and 12 mice in the infection groups. At day 1, mice were subcutaneously injected in the neck region with 1 mg/kg d-C(1–21) in PBS/cholesterol or with PBS/cholesterol alone. Cholesterol was added 5% v/v, 2 mg/ml in ethanol. The peptide and control groups were blinded to avoid any influence by the researchers. After 24 h (group 1) or after 7 days (group 2), mice were intraperitoneally infected with 10^7^ CFU *S. suis* P1/7 in THB or with THB alone as control. 24 h after infection, a few drops of blood were collected via cheek puncture for bacterial count. During the infection phase of the experiment, mice were checked every 12 h in the acute phase of disease (first 48 h) and thereafter daily until the end of the study. A cumulative clinical score was given to the mice as measure of disease using several parameters as depicted in Table 4, according to Seitz *et al.* [31]. When a mouse obtained a clinical score of 2 for a minimum of 3 out of 8 scoring points two days in a row, or in case of severe weight loss (>20%), the mouse was euthanized for animal welfare reasons (humane end point (HEP)) and the organs were collected for bacterial counts as described hereafter. Seven days post infection all mice were sacrificed for further analysis. Mice were anesthetized using isoflurane and 1 mL blood was drawn via heart puncture, followed by cervical dislocation. The peritoneum was flushed with 5 mL PBS/0.5 mM EDTA and diluted in 10 mL ice cold PBS/0.5% FCS. Spleen, lungs, liver, lymph nodes (axillary, inguinal, and mesenteric), brain, kidney and bone marrow were collected and stored in ice cold PBS. All organs, except the bone marrow and lymph nodes were weighed using a Sartorius microbalance. The peritoneal lavage (PTL) samples were counted using the Countess II Automated Cell Counter (Invitrogen, CA, USA). The lungs, liver, brain and kidney were meshed through a 40 µm filter (BD Biosciences) with 5 mL PBS to obtain single cell suspensions. Spleen and lymph nodes were digested with digestion buffer (1.5 WU/mL liberase TL grade, 100 Units/ml recombinant DNAse I, both Roche) for 30 min at 37 °C and meshed through a 40 µm filter using 5 mL PBS/0.5 mM EDTA. The red blood cells of the blood and spleen were lysed using an isotonic ammonium chloride buffer (155 mM NH_4_Cl, 10 mM KHCO_3_, 0.1 mM EDTA) for 5–10 min on ice, washed 1x with PBS and were resuspended in FACS buffer (PBS/0.5% BSA). Bone marrow samples were flushed out the femur and tibia of both legs with 5 mL PBS and filtered through a 40 µm filter. A sample of the blood, bone marrow, spleen, peritoneal lavage and lymph nodes was taken and stained for 30 min with different antibody panels (Table 1) and measured using the BD FACS Canto-II and analyzed with FlowJo V8. Of the lungs, liver, brain, kidney, spleen and peritoneal lavage samples, a 10-fold serial dilution was prepared and the samples were plated on TSA plates containing 5% (v/v) defibrinated sheep blood. The colonies were allowed to grow for 48 h at 37 °C. The number of colonies were counted, with ≤ 100 CFU/mL (2 log CFU/mL) as detection limit of the assay and calculated as CFU/mg organ.

### Ethics statement

All mice were kept under specific pathogen-free conditions with free access to food and water under the guidelines for animal experimentation as approved by the Dutch central authority for scientific procedures on animals (CCD, License number: AVD108002015175).

### Statistics

Samples were compared to no-peptide-controls using two-way ANOVA with the Dunnett post-hoc test. Samples were paired for cell culture samples. *=p ≤ 0.05; **=p ≤ 0.01; ***=p ≤ 0.001; ****=p ≤ 0.0001.

## Results

### d-CATH-2 and its derivatives efficiently kill several *S. suis* type 2 strains in both THB and RPMI + FCS

**Fig. 1:**
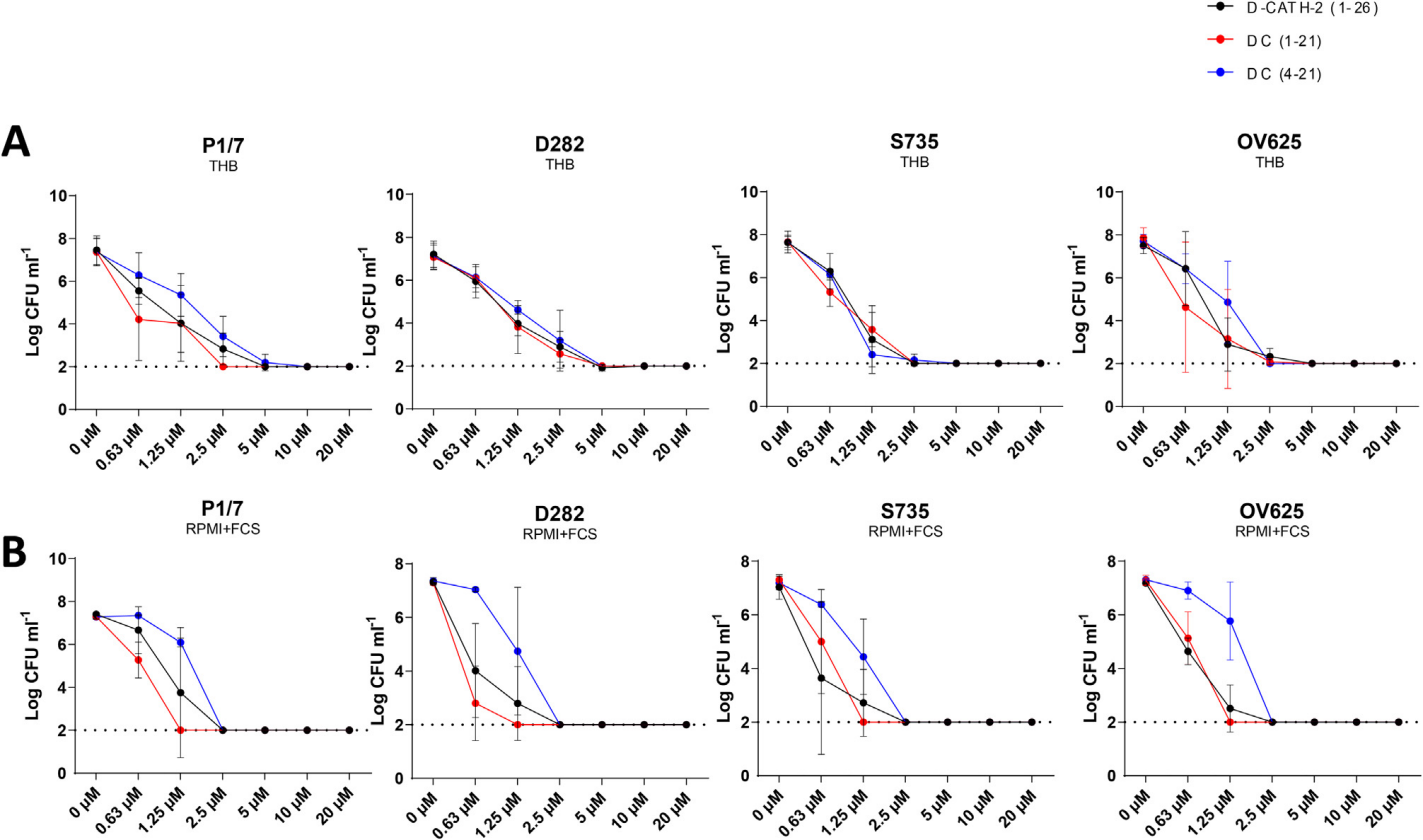
d-CATH-2 and its derivatives efficiently kill several *S. suis* type 2 strains in both THB and RPMI + FCS. Antibacterial activity of d-CATH-2 and its derivatives against 10^6^ CFU/mL *S. suis* type 2 strains (P1/7, D282, S735, and OV625) was tested using a colony count assay in both THB medium **(A)** and RPMI + FCS **(B)**. 2 log CFU/mL was set as detection limit for the experiment. Data is plotted as average +/- SEM (N = 3–4).

Antimicrobial activity of d-CATH-2 and its derived peptides was assessed against 4 different *S. suis* serotype 2 strains. The MBC of the three peptides for the *S. suis* strains was 2.5–5 µM in THB medium (Fig. 1**A** and Table 2). However, most of the subsequent assays were performed in cell culture medium RPMI + 10% FCS, which contains serum proteins and cationic ions that can influence the activity of cathelicidins [32], [33]. Therefore, the MBC of d-CATH-2 and its derived peptides was also tested in RPMI + 10% FCS medium. The MBC of d-CATH-2 and d-C(1–21) slightly increased to 0.6–2.5 µM, whereas the MBC of the shortest peptide, d-C(4–21), remained stable at 2.5 µM (Fig. 1**B** and Table 2).

**Table 2:** MBC values of d-CATH-2 against *S. suis* strains.

	THB			RPMI + 10% FCS		
	d-CATH2	dC(1–21)	dC(4–21)	d-CATH2	dC(1–21)	dC(4–21)
P1/7	2.5–5	2.5	5–10	1.25–2.5	1.25	2.5
S735	1.25–2.5	2.5	2.5	0.6–2.5	1.25	2.5
D282	2.5–5	2.5–5	2.5–5	0.6–2.5	0.6–1.25	1.25–2.5
OV625	1.25–5	0.6–5	1.25–2.5	1.25–2.5	1.25	2.5

MBC values for the different peptides depending on the bacterial strain and the medium.

### d-CATH-2 and its derivatives inhibit LTA-SA- or *S. suis*-induced immune cell activation by binding to LTA

Natural (L-)CATH-2 is known to inhibit LPS- and LTA-induced activation of a murine macrophage cell-line [9]. However, it is unclear whether the all d-enantiomer of CATH-2 is also capable of inhibiting LTA-induced activation of primary cultured murine BMDMs and BMDCs. In addition, cathelicidins can in some instances be cytotoxic to mammalian cells at higher concentrations [32]. Therefore, murine BMDMs and BMDCs were exposed to d-CATH-2, d-C(1–21) and d-C(4–21) added at either day 1 or day 6 of culture, to observe effects of the peptides on both cell viability and differentiation.

BMDMs were relatively sensitive to addition of d-CATH-2 and its derivates, especially to d-C(1–21) (Figure S1**A**), showing a marked reduction in metabolic activity at 5 µM. BMDCs had some reduced viability, starting from 2.5 µM peptide, with no difference between the three peptides (Figure S1**B**). Both BMDMs and BMDCs were less sensitive if the peptides were added at day 1 of the culture, with only a small reduction in viability at 5 µM **(**Fig S1**C and D**), although a similar slight reduction in viability was seen for d-C(1–21). Flow cytometry analysis also showed a decrease in BMDM purity, with a lower percentage of cells expressing the macrophage marker F4/80. Those BMDMs that did survive had an increased F4/80 expression and reduced MHC-II (Figure S1**E**). A minor reduction in live BMDCs is only visible at 5 µM, without affecting the other cell markers (Figure S1**F**).

**Fig. 2:**
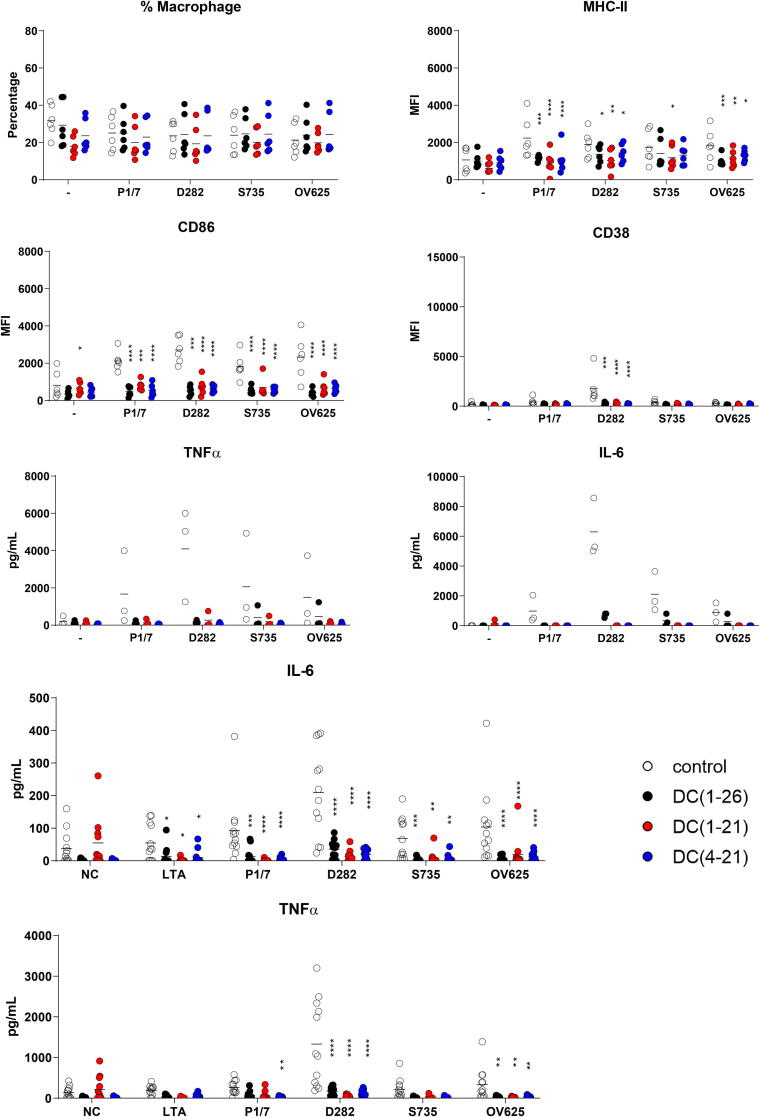
d-CATH-2 and its derivatives inhibit LTA-SA- or *S. suis*-induced activation. Mouse BMDM cells were cultured for 6 days before they were activated with different *S. suis* serotype 2 strains at an MOI of 0.2. Bacteria were mixed for 5 min with 1.25 µM d-CATH-2 or its derivatives before stimulation. After 24 h of stimulation, cells were analyzed by flowcytometry plotting the median fluorescence index (MFI) **(A)** and cytokine expression was measured **(B)**. 5*10^5^ splenocytes, freshly isolated from WT mice using a digestion buffer followed by filtering through a 40 µM cell filter, were activated with different *S. suis* type 2 strains premixed with 5 µM d-CATH-2 or its derivatives at an MOI of 0.2. After 24 h of stimulation, secreted cytokines were measured using ELISA **(C)**. Data is plotted as average +/- SEM (N = 3–6). Samples were compared to no-peptide-controls using two-way ANOVA with the Dunnett post-hoc test. Samples were paired for cell culture samples. *=p ≤ 0.05; **=p ≤ 0.01; ***=p ≤ 0.001; ****=p ≤ 0.0001.

To analyze the effect of peptides on bacterial stimulation of macrophages, four different strains of *S. suis* serotype 2 were mixed with 1.25 µM peptide and added to BMDMs at day 6 of culture. Stimulation of BMDMs with peptide did not influence the percentage of live macrophages in culture, as shown by flow cytometry. Bacterial stimulation of BMDMs showed a typical upregulation of activation markers, like MHC-II, CD86 and CD38. However, this upregulation was strongly inhibited by all three peptides for all four bacterial strains (Fig. 2**A**). Similar results were found for BMDCs (Figure S2**A**). In addition, the secretion of TNFα and IL-6 seemed to be lowered in the presence of the peptides, although the results were not significant (Fig. 2**B**). To study the influence of the peptides on *S. suis*-induced activation in a more complex system, total splenocytes from mice were activated *ex vivo*. Besides live *S. suis* bacteria, purified LTA was used for activation. In the presence of peptides, neither LTA nor whole *S. suis* bacteria were able to activate the splenocytes, shown by the inhibition of TNFα and IL-6 secretion (Fig. 2**C**).

**Fig. 3:**
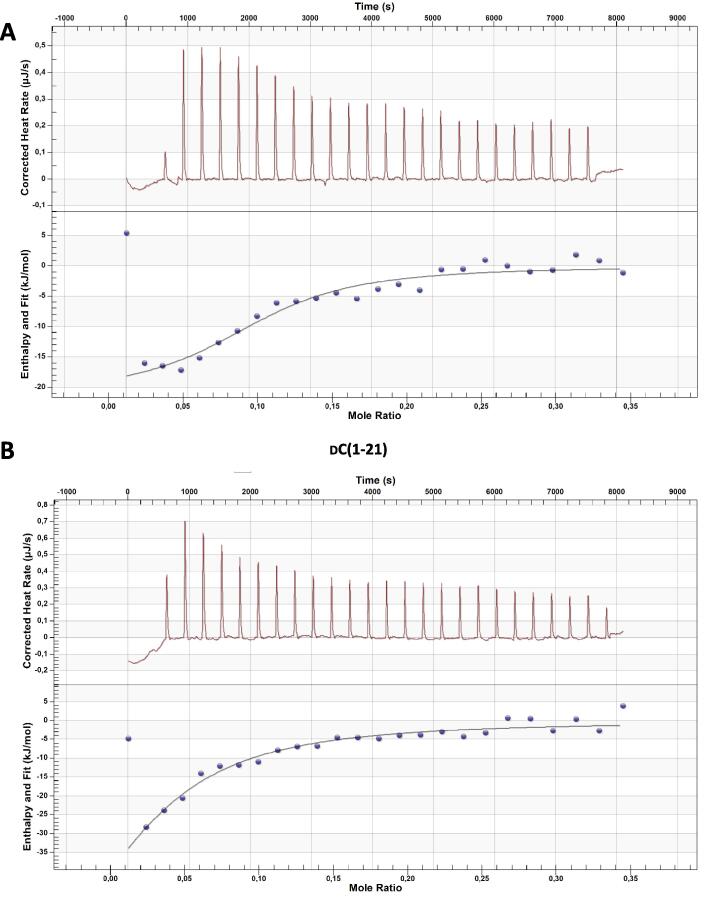

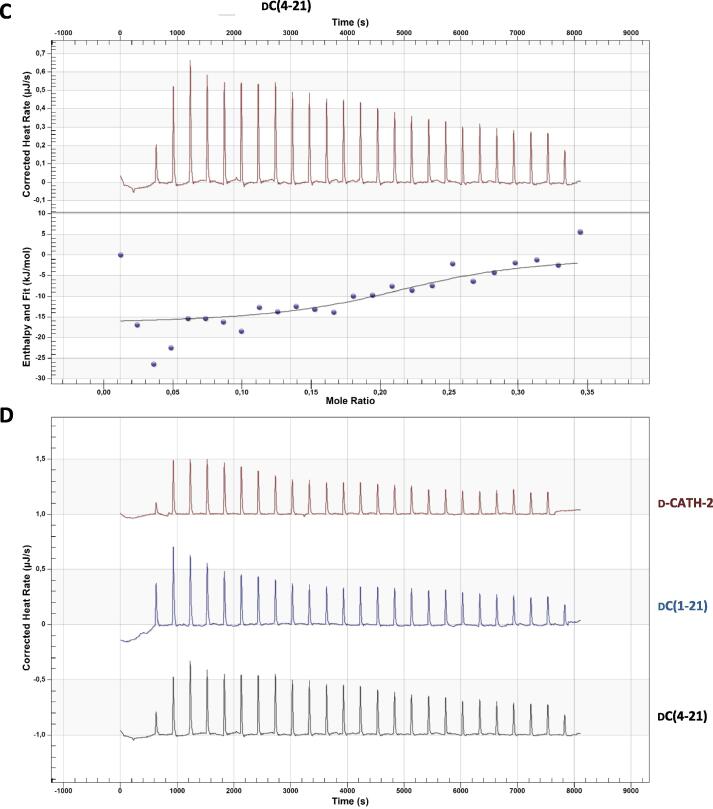
d-CATH-2 and its derivatives bind to LTA. Thermodynamic binding capacity of 200 µM d-CATH-2 **(A)**, d-C(1–21) **(B)**, and d-C(4–21) **(C)** to 37.2 µM LTA-SA was measured using isothermal titration calorimetry (ITC). Every 300 s, 1.99 μL peptide solution was titrated into 164 μL LTA solution. The corrected heat rate (µJ/sec) is plotted (top panel) and normalized integrated heat was plotted against the molar ratio between LTA and the peptide (lower panel). Experiments (N = 3) were averaged before plotting and fitting an independent model. The corrected heat rate of d-CATH-2, d-C(1–21), and d-C(4–21) is depicted for comparison **(D)**.

To study whether the inhibitory effect on activation by the peptides was due to a direct interaction of the peptides with LTA, the LTA binding capacity of peptides was tested using isothermal titration calorimetry (ITC). All three peptides inhibiting LTA-induced activation showed direct binding to LTA with a dissociation coefficient K_d_ ranging from 2 to 10 µM. Interestingly, d-C(1–21) showed weaker binding compared to the other two peptides, with a higher K_d_ and less peptide binding to one LTA molecule (Fig. 3 **and** Table 3), although the three peptides are equally efficient in inhibiting LTA- and *S. suis-*induced activation.

**Table 3:** ITC data.

	d-CATH-2	dC(1–21)	dC(4–21)
K_d_ (µM)	3.039	10.22	2.12
n	0.543	0.207	1.209
ΔH (kJ/mol)	−21.16	−85.64	−16.73
ΔS (J/mol·K)	37.39	−180.6	54.68

Overview of ITC results of the binding capacity of 200 µM d-CATH-2, dC(1–21) or d-C(4–21) to 37.2 µM LTA-SA. K_d_ – dissociation coefficient (µM); n – number of peptide molecules binding to one LPS molecule; ΔH – enthalpy changes; -ΔS – entropy changes.

### d-CATH-2 and its derivatives increase BMDM culture efficiency

**Fig. 4:**
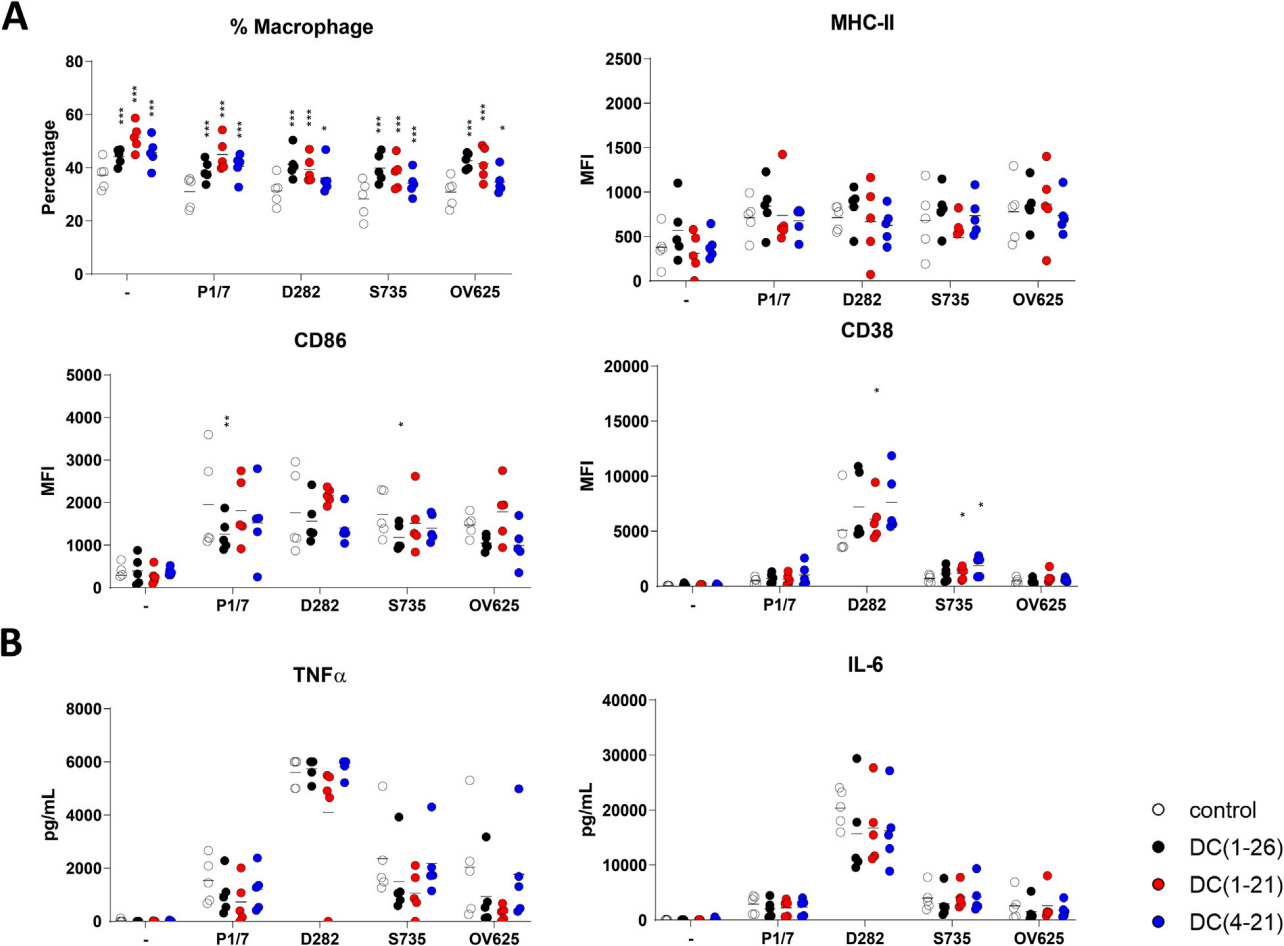
d-CATH-2 and its derivatives increase BMDM efficiency and slightly enhance the activation by *S. suis* serotype 2 strains. Mouse BMDM cells were cultured for 6 days. At day 1, 1.25 µM d-CATH-2 or its derivatives were added for 24 h. At day 6 the cells were activated with different *S. suis* type 2 strains at an MOI of 0.2. Bacteria were mixed for 5 min with 1.25 µM d-CATH-2 or its derivatives before stimulation. After 24 h of stimulation, cells were analyzed by flowcytometry showing median fluorescence index (MFI) **(A)** and cytokine expression was measured **(B)**. Data is plotted as average +/- SEM (N = 3–6). Samples were compared to no-peptide-controls using two-way ANOVA with the Dunnett post-hoc test. Samples were paired for cell culture samples. *=p ≤ 0.05; **=p ≤ 0.01; ***=p ≤ 0.001; ****=p ≤ 0.0001.

To further study the effect of d-CATH-2 and its derivatives on macrophages, cells were exposed to the peptides at day 1 of culture for 24 h. The differentiation efficiency of the BMDMs was enhanced by the early exposure of the peptides, shown by a higher percentage of cells expressing F4/80 at day 6, which was most pronounced for d-C(1–21) (Fig. 4**A**). However, the activity of the cells was not changed based on levels of the activation markers MHC-II, CD86 and CD38 (Fig. 4**A**), nor was there any difference in cytokine expression by the peptide treated cells compared to non-treated cells (Fig. 4**B**).

Similar results were found in the BMDC culture when exposing the cells to the peptides on day 1 of the culture for 24 h. Although the percentage of BMDCs at day 7 did not change, there was also no difference in the expression of the activation markers. However, the macrophage marker F4/80 was increased, indicating a slight skewing towards macrophage like cells (Figure S2**B**).

### d-C(1–21) reduces the clinical symptoms of *S. suis* P1/7 in mice

**Fig. 5:**
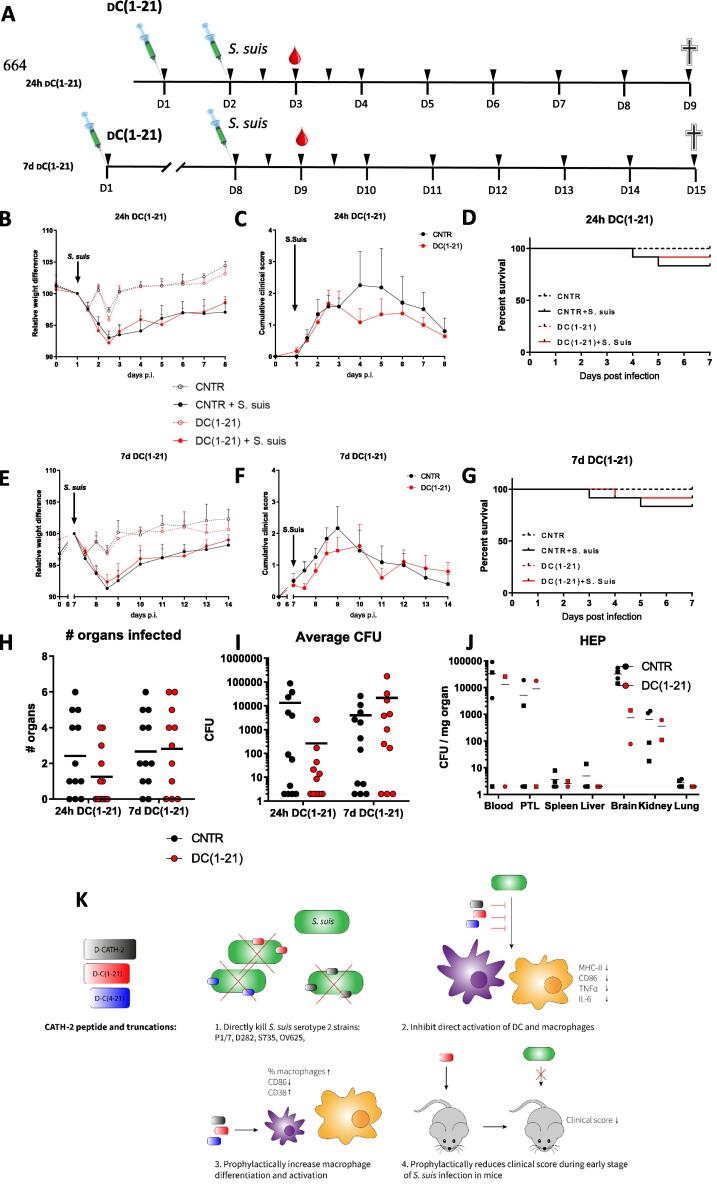
Prophylactic d-C(1–21) s.c. injection reduces the clinical symptoms of *S. suis* P1/7 in mice. Shown is a schematic overview of the *in vivo* experimental set up. At day 1, all mice were subcutaneously injected with d-C(1–21) or a control in the neck region. Either after 24 h (24 h d-C(1–21)) or 7 days (7 d d-C(1–21)) the mice were intraperitoneally injected with 10^7^ CFU *S. suis* P1/7 or only THB. Twenty-four hours after infection, a few drops of blood were collected and at 7 days post infection the mice were sacrificed for analysis. The black arrows indicate the moment of animal welfare evaluation by weighing and score for clinical symptoms **(A)**. The relative weight difference is depicted for 24 h d-C(1–21) **(B)** and 7 d d-C(1–21) **(E)**. The cumulative clinical score of 8 different parameters is shown for 24 h d-C(1–21) **(C)** and 7 d d-C(1–21) **(F).** Survival curves are shown and bacterial counts in different organs of mice reaching HEP are depicted for 24 h d-C(1–21) **(D)** and 7 d d-C(1–21) **(G)**. The number of organs per mouse in which *S. suis* bacteria were found **(H)** and the average CFU per organ per mouse **(I)** is shown for both groups. The bacterial burden in organs of mice which reached the HEP before the end of the study, with circles depicting mice infected 24 h post peptide injection and squares depicting mice infected 7 days post peptide injection **(J)**. Results are depicted as mean +/- S.E.M. (CNTR n = 4, CNTR + *S. suis* n = 12, d-C(1–21) n = 4, and d-C(1–21) + *S. suis* n = 12). (**K**) Results of the paper are graphically summarized.

Previously, our group has shown that *in ovo* injection of d-CATH-2 three days before hatch protects chickens for seven days post hatch against infection [24]. Since addition of d-CATH-2 both enhanced the efficiency of the murine BMDM culture and balanced the inflammatory response, we questioned whether injection of d-C(1–21) could boost the immune response in mice as well. Therefore, mice were injected subcutaneously with 1 mg/kg d-C(1–21) at day 1 and were subsequently infected with 10^7^ CFU/mL *S. suis* P1/7 intraperitoneally either 24 h or 7 days post peptide injection. Mice were weighed twice a day during the acute phase of infection, and then daily until 7 days post infection (Fig. 5**A**). Both peptide-treated mice and control mice lost approximately 8% bodyweight up to 48 h post infection, then started to gain weight again, in both the 24 h (Fig. 5**B**) or 7 d post peptide injection groups (Fig. 5**E**). In addition, a cumulative clinical score was given twice a day during the acute phase of infection and daily during the chronic phase to the mice using a scoring table (Table 4). A small reduction of cumulative clinical score for mice was shown in the late stage of disease, if mice were infected 24 h post peptide injection (Fig. 5**C**). Similarly, if infected 7 days post peptide injection a reduction in cumulative clinical score was visible at the acute phase of disease (Fig. 5**F**). In addition, treated mice had a higher chance of survival when infected either 24 h (Fig. 5**D**) or 7 days post peptide injection (Fig. 5**G**).

**Table 4:** Clinical scoring parameters for cumulative scoring of *S. suis*-infected mice.

	Score		
	0	1	2
Body weight	Constant or gain	>5% weight loss	> 20% weight loss
Coat	Flat and glossy	Rougher	Bloated
Breathing	Rhythmic	Rapid	Rapid and abdominal
Dehydration	Normal skin elasticity	Reduced skin elasticity	Persisting skin fold
Bearing	Normal	Curved back	Huddled
Eyes	Normal	Moderately squeezed	Squeezed and swollen
activity	Normal	Reduced activity	Apathy
locomotion	Normal	Reduced coordination	Unsteady, apraxia

The cumulative clinical score was defined as the sum of the clinical scoring for eight parameters. Mice were euthanized for animal welfare reasons at HEP when they endured severe clinical signs (defined as: 2 days in a row a score of 2 on 3 of the 8 points) or in case of severe weight loss (>20%).

Bacterial counts in the different organs were determined as well. Twenty-four hours post infection, all mice, treated or not, had *S. suis* bacteria in the bloodstream at a level of 10^5^-10^6^ CFU/mL (Figure S3**A**). After 7 days, most mice were able to deplete all bacteria from the blood, which was more efficient in 24 h d-C(1–21) treated mice compared to untreated mice, where no mice had bacteria left in the bloodstream (Figure S3**B**). More cells were present in the peritoneal lavage after infection, but not different for treated or untreated mice (Figure S3**C**), nor were differences in specific cellular subsets found with flow cytometry (data not shown). Moreover, most mice were able to clear the bacteria in the peritoneum at similar rates in the treated and untreated groups (Figure S3**E**). The spleens of *S. suis* infected mice were enlarged, showing that the infection model was efficient at inducing a systemic immune response, however, no differences in spleen size were found between peptide treated and untreated mice (Figure S3**D**). Only minor differences were found in the number of *S. suis* present in different organs (Figure S3**E**). However, counting the number of organs in which bacteria could be found, showed that 24 h d-C(1–21) treated mice had generally less *S. suis* positive organs (Fig. 5**H**) and lower total CFU counts (Fig. 5**I**) compared to the untreated mice, although these results were not statistically significant. Moreover, the lower bacterial counts were not found for 7 day d-C(1–21) treated mice. In addition, d-C(1–21) treated mice reaching the HEP before the end of the study showed less bacterial counts, especially in the brain, pointing towards a less severe course of disease (Fig. 5**J**). The immune cells were analyzed for the different organs; however, no differences were found between treated and untreated mice (Figure S4**A-C**).

## Discussion

Cathelicidins have both an antimicrobial as well as an immunomodulatory function. Due to this dual function combined with the aspecific and non-proteinaceous molecular target of the peptides, bacteria are less likely to develop resistance and therefore cathelicidins are an interesting candidate as alternative to antibiotics. Chicken CATH-2 has been previously shown to have a good antibacterial activity against a wide variety of bacterial strains [32], [34], [35], [36]. However, the activity against the Gram-positive encapsulated *S. suis* was, up to now, unknown. *S. suis* is a porcine commensal bacterium which can also cause invasive infections in pigs [25] and sepsis and meningitis in humans [26]. Of the 35 known different serotypes, serotype 2 is found to be the most common cause of *S.* suis infection in pigs and humans [28]. Therefore, in this study, we investigated the possibility to use chicken CATH-2 as an alternative to antibiotics for *S. suis* infection. To enhance the stability of cathelicidins *in vivo*, the l-amino acids were substituted by d-amino acids to prevent proteolytic cleavage [37]. In addition, two shorter d-CATH-2 variants were designed.

The full d-enantiomer d-CATH-2 and both its shorter derivatives showed an equally strong killing capacity of four clinically relevant strains of *S. suis* serotype 2 in bacterial medium. However, the salt concentration of the assay medium can strongly influence the activity of cathelicidins. Most cathelicidins lose antibacterial activity at increasing cation concentrations, which is especially important *in vivo* [38], [39], [40]. Increasing salt concentrations can influence the secondary structure of α-helical cathelicidins, which may lead to loss of activity [41]. In addition, other factors such as the presence of serum can reduce their activity, possibly to protect mammalian cells from collateral damage during infection [42]. However, increased activity was also found for many peptides in the presence of serum containing culture medium depending on the bacterial strain [9]. Therefore, the MBC of d-CATH-2 and both its shorter derivatives was also tested in culture medium containing 10% FCS. The antibacterial activity of d-CATH-2 and d-C(1–21) against the four tested *S. suis* strains was slightly enhanced in culture medium, whereas d-C(4–21) appears to be more sensitive to increased salt or serum concentrations, which makes the shortest peptide slightly less favorable as direct antimicrobial drug candidate. However, as this study focuses only on serotype 2 strains, the peptide might still be suitable as antimicrobial for other serotypes [28], [43]. In the immunomodulation experiments there was little to no difference between any of the tested enantiomers. Compared to full length, d-C(1–21) reduced the MHC-II expression of DCs when stimulated with several serotypes, and slightly reduced CD38 expression. Conversely, the full length peptide reduced CD86 expression when administered during culture, whereas the truncations had no effect. The differences observed between the various enantiomers were overall mild. Therefore, we selected d-C(1–21) as the most promising peptide, as it has the most potent antimicrobial activity, equivalent immunomodulatory activity compared to the other peptides, and is slightly shorter than the full length peptide.

Inhibition of LPS-induced, and to a lesser extent LTA-induced activation, is a widely studied immunomodulatory aspect of cathelicidins. In this study, LTA-induced activation was strongly inhibited by all three peptides and this can be partially explained by direct binding to LTA. The dissociation coefficient to LTA is with 2–10 µM much higher than previously shown for l-CATH-2 to LPS with a dissociation coefficient of 0.08 µM, [32] indicating a stronger binding to LPS than to LTA. However, since d-CATH-2 was highly efficient at inhibiting LTA-induced activation, it might also be that the inhibition of LPS- and LTA-induced activation of macrophages occur with different mechanisms. In addition, in this study LTA from *S. aureus* was used to induce TLR-2 activation, while *S. suis* LTA by itself does not potently induce TLR-2 dependent activation in DCs [44]. It is therefore likely that the inhibition of *S. suis* induced activation observed is through another mechanism, potentially by binding to other lipoproteins.

In addition to inhibition of LTA-induced activation, d-CATH-2 and its derivatives strongly inhibit *S. suis*-induced activation when bacteria are killed by peptides prior to activation of the cells. This inhibition is as efficient for macrophages as for dendritic cells, without affecting cell markers and relative cell numbers. This silent killing was also found for *E. coli*, [45] *P. aeruginosa*, [34], [46] and *S. aureus*, [34], [47] when bacteria were killed by l-CATH-2, but not by exposure to heat or antibiotics. Inhibition was also not observed if the peptide concentration remains below bactericidal concentrations. This viability-dependent regulation of immune activation by cathelicidins balances the strength of the immune response, prevents overactivation by killed bacteria and thereby reduces the collateral damage of an unrequired immune response.

Since d-CATH-2 and its derivatives strongly inhibited LTA-induced activation, improved the efficiency of macrophages and efficiently and silently killed the four *S. suis* strains, the next step was to study the possible protective effect of the peptides *in vivo*. Previously, it was shown that *in ovo* administration of d-CATH-2 protects chickens up to 7 days post hatch from *E. coli* infection, [24] suggesting immunomodulation by the peptide as the main mechanism of protective action. In this study injection of d-C(1–21) reduced disease severity and increased the survival rate, although the differences are relatively small compared to the *in ovo* experiments mentioned above. It is unclear if this difference related to species difference where these peptides may have less activity in mammalian models. However, the human cathelicidin LL-37 has been proven to be beneficial in infection studies in mice before. Intravenous administration of LL-37 in septic mice improved the survival and reduced the bacterial load in the blood and peritoneum [48], [49]. However, LL-37 was administered either just before [48] or immediately after [49] induction of sepsis. Still, these models most likely show not only a direct effect of LL-37 on the infection, but also some immunomodulation, since 2 µg LL-37 per mouse is too low for solely direct antibacterial killing. In addition, mice are not the natural host for *S. suis*, which results in a relatively high bacterial burden needed to establish infection. The prophylactic effects in pigs could be much higher and should still be investigated. Moreover, in such a model, more detailed parameters such as colonization efficiency in the intestine and tonsils could be measured, which provides a much more complete overview of effects that are important *in vivo.*

Another improvement in activity of d-CATH(1–21) in our model might be achieved by the timing of peptide administration. Administration during embryonic phase would resemble the *in ovo* administration better, but embryonic peptide administration will be difficult and unethical in mammalian farm animals. A possible solution might be to administer a hormonally active form of vitamin D (1,25(OH)2D) to the mother. This has been shown to increase the placental cathelicidin expression, [50] which might reduce the risk of infection [51]. Lastly, the bacterial species might also explain the difference in protection. It was demonstrated in a wax moth model that a sea snake cathelicidin (HcCATH) protects better against *P. aeruginosa* infection than against *S. aureus* infection [52]. However, more research should be performed to improve the understanding about the immunomodulatory functions of cathelicidins.

## Conclusion

Taken together, this study showed a direct antibacterial effect of chicken cathelicidin-2 (d-CATH-2) and its shorter derivatives on different *S. suis* strains as well as an immunomodulatory effect *in vitro* by skewing bone marrow-derived dendritic cells towards a more macrophage-like phenotype. In addition, the peptides are able to reduce the inflammatory response *in vitro* for LTA and live *S. suis* bacteria. The immunomodulatory effect *in vivo* is present, but should be further optimized to improve d-CATH-2 for potential clinical use.

## Declaration of Competing Interest

The authors declare that they have no known competing financial interests or personal relationships that could have appeared to influence the work reported in this paper.
