# Supplementary material for: d-enantiomers of CATH-2 enhance the response of macrophages against Streptococcus suis serotype 2
Source: J Adv Res. 2021 May 26;36:101–12. doi: 10.1016/j.jare.2021.05.009 (PMC8799869; doi:10.1016/j.jare.2021.05.009)
Supplement: Supplementary data 1 [file mmc1.docx]

**d-enantiomers of CATH-2 enhance the response of macrophages against *Streptococcus suis* serotype 2**

*Roel M. van Harten, ^a^ Johanna L.M. Tjeerdsma-van Bokhoven, ^a^ Astrid de Greeff, ^b^ Melanie D. Balhuizen, ^a^ Albert van Dijk, ^a^ Edwin J.A. Veldhuizen, ^a*^ Henk P. Haagsman,^a^ Maaike R. Scheenstra ^a^*

^a^Division of Infectious Diseases and Immunology, Department of Biomolecular Health Sciences, Utrecht University, Utrecht, The Netherlands

^b^Wageningen Bioveterinary Research, Wageningen University & Research, Houtribweg 39, 8221 RA Lelystad, The Netherlands

**Supplementary Files**
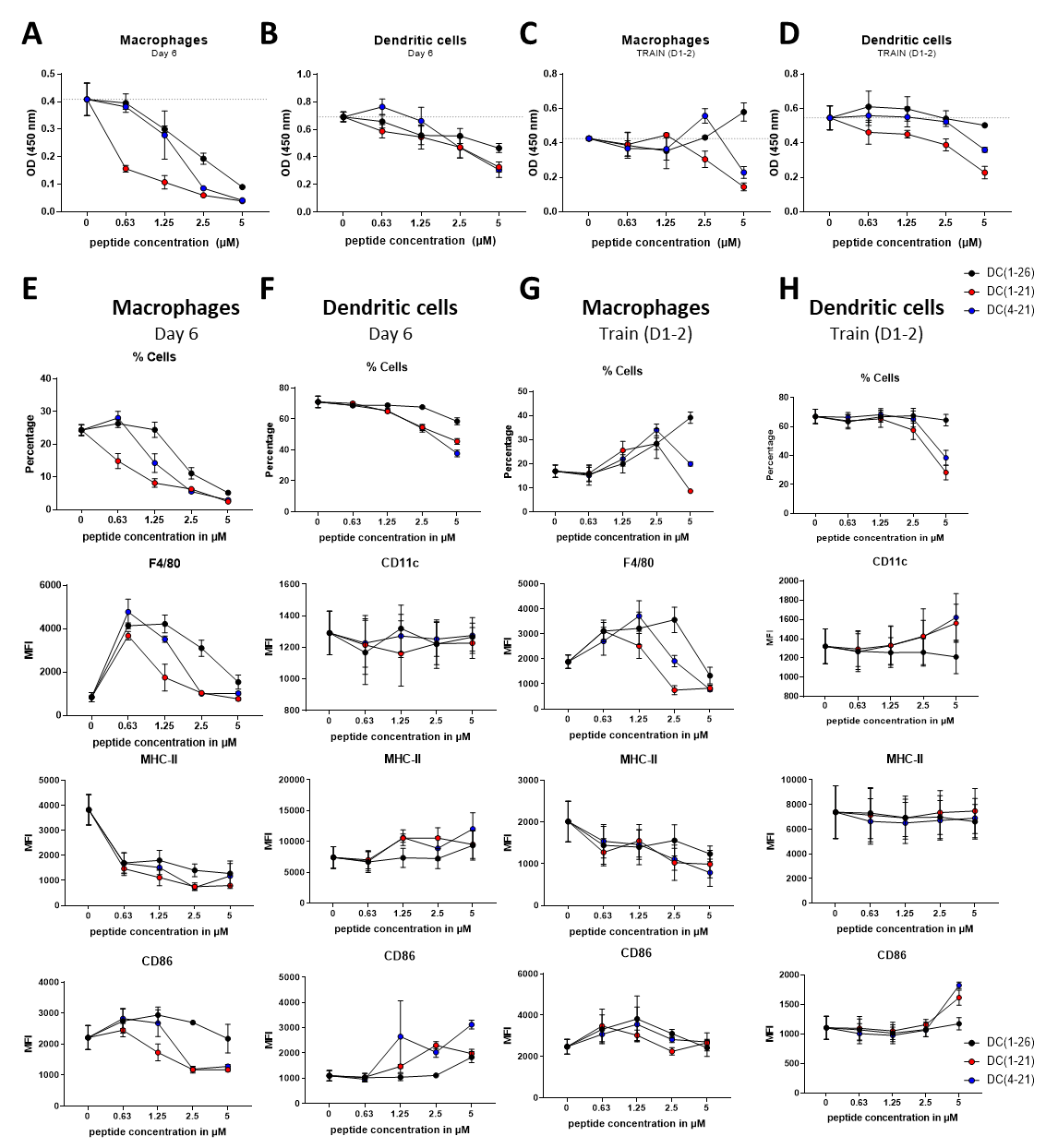


**Supplementary figure 1. Peptide titration on BMDM and BMDCs for cytotoxicity.**

Mouse BMDM **(A, C, E, G)** and BMDCs **(B, D, F, H)** were cultured for 6 days with GM-CSF and M-CSF respectively. Different concentrations were added at day 6 **(A, B, E, F)** or the cells were primed with different concentrations peptides at day 1-2 **(C, D, G, H)**. At day 7, cell viability was tested using WST-1 reagent, with no-peptide-control set to 100% viability (shown as a dashed line) **(A-D)** and cells were analyzed by flow cytometry for cell marker expression **(E-H)**. Results are depicted as mean +/- S.E.M. (n=3).


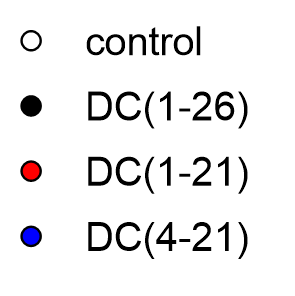

**Dendritic cells**

*Train D1-2*

**Dendritic cells**

*Stimulation + peptide*

**A**

**B**

**Supplementary figure 2. Dendritic cell primed by peptides have increased macrophage markers.**

Mouse BMDCs were cultured for 6 days with M-CSF and either primed with 1.25 µM peptides at day 1-2 **(A)** or 1.25 µM peptide was added at day 6 during stimulation **(B)**. Cells were analyzed by flowcytometry for cell marker expression **(C, D)**. Results are depicted as mean +/- S.E.M. (n=6).

24h

7d

**A**

**B**

**E**

**C**

**D**

**Supplementary figure 3. Bacterial counts in the blood and different organs of *S. suis* infected mice.**

Blood was drawn via cheek puncture 24h after infection **(A)** and via heart puncture after 7 days **(B)** and plated on TSA/5% sheep blood plates for bacterial counts **(B)**. The peritoneum was flushed at day 7 and cells present in the peritoneal lavage (PTL) were counted **(C)**. The spleens were weighed **(C)**. The single cell suspension of the different organs was plated on TSA/5% sheep blood plates for bacterial counts. CFU per mg organs was calculated **(E).**


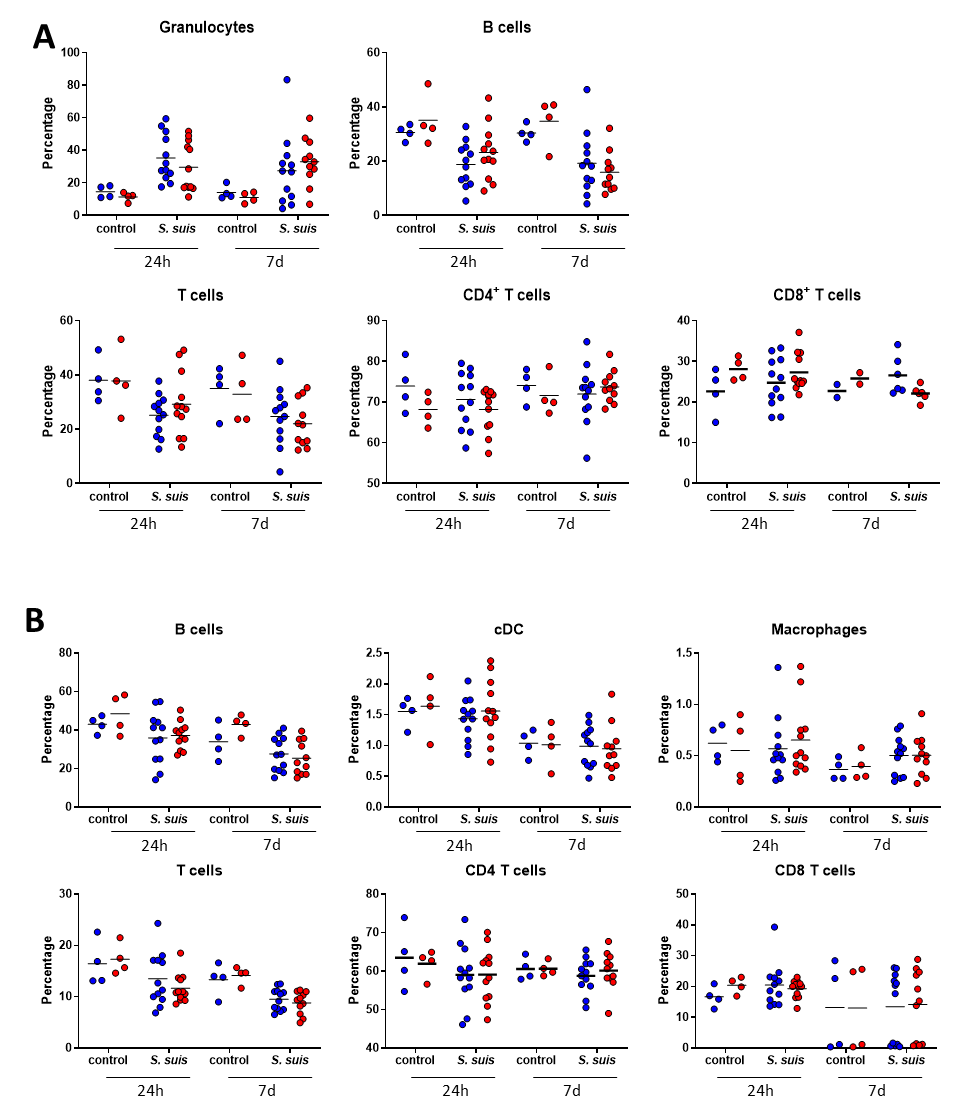

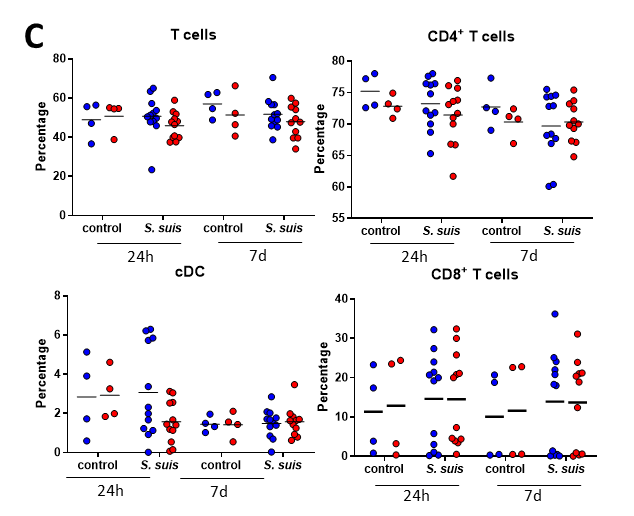


**Supplementary figure 4. Flow cytometry analysis of *S. suis* infected mice.**

Mice were sacrificed 7 days post infection and fully analyzed. Single cell suspensions of the organs were prepared and analyzed using flow cytometry. A selection of the results of the blood **(A)**, spleen **(B)**, and the axillary lymph nodes **(C)** are depicted.
